# PROPERTY: study protocol for a randomized, double-blind, multicenter placebo-controlled trial assessing neurotoxicity in patients with metastatic gastrointestinal cancer taking PHYCOCARE® during oxaliplatin-based chemotherapy

**DOI:** 10.1186/s13063-023-07071-z

**Published:** 2023-01-20

**Authors:** Christele Le Gouill-Jaijarat, Yann Péréon, Maxime Leroy, Olivier Lépine, Aymeric Loloum, Claire Peluchon, Christelle Volteau, Anne-Sophie Martineau, Simon Korner, Caroline Perrault, Asmahane Benmaziane, Paul Girot, Caroline Petorin, Clément Perret, Catherine Ligeza-Poisson, Didier Mayeur, Laurent Flet, Anne Chiffoleau, Alexandra Poinas, Jaafar Bennouna

**Affiliations:** 1 Gastroenterology Department, CHU Nantes (Nantes Teaching Hospital), Nantes Université, Nantes, France; 2 Department of Clinical Neurophysiology, Reference Centre for Neuromuscular Diseases AOC, Filnemus, Euro-NMD, CHU Nantes, Nantes Université, Place Alexis-Ricordeau, Nantes, France; 3 Sponsor Department, Nantes Université, CHU Nantes, Nantes, France; 4 AlgoSource, Saint-Nazaire, France; 5 Clinical Investigation Centre CIC1413, Nantes Université, CHU Nantes, Inserm, Nantes, France; 6 Medical Oncology Department, Hôpital Foch, Paris, France; 7 Gastroenterology Department, CHD Vendée, La Roche sur Yon, France; 8 CHU Estaing, Clermont-Ferrant, France; 9 Center Hospitalier Privé Saint-Grégoire, Rennes, France; 10 Clinique Mutualiste de l’Estuaire Saint-Nazaire, Saint-Nazaire, France; 11 Centre Georges et François Leclerc, Dijon, France; 12 Department of Pharmacy, CHU Nantes, Nantes Université, Nantes, France

**Keywords:** Chemotherapy-induced peripheral neuropathy, Sensory neuropathy, Neurotoxicity, Spirulina extract, Phycocyanin, Oxaliplatin, Adverse drug reaction, Prophylaxis, Randomized controlled trial

## Abstract

**Background:**

Chemotherapy-induced peripheral neuropathy (CIPN) is one of the most common adverse effects of antineoplastic agents, ranging in prevalence from 19% to over 85%. Clinically, CIPN is a predominantly sensory neuropathy that may be accompanied by motor and autonomic changes of varying intensity and duration. The high prevalence of CIPN among cancer patients makes it a major problem for both patients and survivors, as well as for their health care providers, especially because there is currently no single effective method of preventing CIPN; moreover, the options for treating this syndrome are very limited.

Phycocyanin, a biliprotein pigment and an important constituent of the blue-green algae *Spirulina platensis*, has been reported to possess significant antioxidant and radical-scavenging properties, offering protection against oxidative stress, which is one of the hypothetic mechanisms, between others, of CIPN occurrence.

**Methods:**

Our hypothesis is that phycocyanin may give protection against oxaliplatin-induced neuropathy in the treatment of gastrointestinal cancers. Our trial will be a randomized double-blind placebo-controlled study with 110 randomized patients suffering from metastatic gastrointestinal adenocarcinoma including esogastric, colorectal, and pancreatic cancers. Patients are being followed up in the gastroenterology or oncology departments of seven French hospitals.

**Discussion:**

Due to the neuropathy, patients need to avoid injury by paying careful attention to home safety; patients’ physicians often prescribe over-the-counter pain medications. If validated, our hypothesis should help to limit neurotoxicity without the need to discontinue chemotherapy.

**Trial registration:**

ClinicalTrials.gov NCT05025826. First published on August 27, 2021.

**Supplementary Information:**

The online version contains supplementary material available at 10.1186/s13063-023-07071-z.

## Administrative information

Note: the numbers in curly brackets in this protocol refer to SPIRIT checklist item numbers. The order of the items has been modified to group similar items together (seehttp://www.equator-network.org/reporting-guidelines/spirit-2013-statement-defining-standard-protocol-items-for-clinical-trials/).

**Table:** 

**Title {1}**	Randomized, double-blind, multicenter placebo-controlled study assessing neurotoxicity in patients with metastatic gastrointestinal adenocarcinoma taking phycocyanin or placebo during oxaliplatin-based chemotherapy
**Trial registration {2a and 2b}**	Registration number NCT05025826
**Protocol version {3}**	The updated protocol is version 2 of May 23, 2022.
**Funding {4}**	PROPERTY was supported by AlgoSource, Saint-Nazaire, France
**Author detail {5a}**	Christele Le Gouill-Jaijarat and Claire Peluchon are part of the Gastroenterology Department of CHU Nantes; Claire Peluchon is also part of the Clinical Investigation Centre CIC1413 (INSERM and CHU Nantes), while Alexandra Poinas is solely a member of the CIC. Maxime Leroy, Caroline Perrault, Christelle Volteau, Anne-Sophie Martineau, Simon Korner and Anne Chiffoleau are from the Sponsor Department of CHU Nantes. Olivier Lépine and Aymeric Loloum are part of the AlgoSource laboratory. Jaafar Bennouna Asmahane Benmaziane, Paul Girot, Caroline Petorin, Clément Perret, Catherine Ligeza-Poisson, and Didier Mayeur are principal investigators, who are members of the Gastroenterology Department of Foch Hospital, Vendée Departmental Hospital, Estaing Hospital, Saint-Grégoire Private Hospital, Saint-Nazaire Hospital, and Georges and François Leclerc Center respectively. Laurent Flet works in the Pharmacy Department and Yann Péréon in the Department of Clinical Neurophysiology, both at CHU Nantes.
**Name and contact information for the trial Sponsor {5b}**	The Sponsor Department of CHU Nantes responds to any requests sent to this e-mail address: BP-direction-de-la-recherche@chu-nantes.fr.
**Role of the Sponsor {5c}**	All the submissions/declarations were made by the Sponsor Department at CHU Nantes, which manages the quality of the data collected. The data collected during the study will be processed electronically in accordance with the requirements of the CNIL (the French Data Protection Authority) and with the European and French regulations on safety matters. The sponsor should address any requests for substantial modifications of the protocol to the French regulatory authorities and/or the relevant Ethics Review Board for approval or notification, in compliance with French Law 204-806 of August 9, 2004 and its implementing decrees. It should be noted that the funding body is not connected with the data collected or with the protocol and any amendments thereto.

## Introduction

### Background and rationale {6a}

Gastrointestinal (GI) cancers account for 26% of the overall incidence of cancer and 35% of cancer-related deaths [1]. They mainly include esophageal and gastric carcinoma, colorectal carcinoma, pancreatic carcinoma, and hepatocarcinoma [2]. The drugs used most commonly as first-line therapy are platinum derivatives (cisplatin, oxaliplatin), 5-fluorouracil (5-FU), and other pyrimidine analogs (capecitabine), either administered as single agents or, frequently, in combination [2].

As with other chemotherapy drugs, the basic cytotoxic effect of platinum-based compounds (DNA damage) is not restricted to tumor cells, thus resulting in a variety of adverse effects.

Neurotoxicity in response to platinum-based therapy is the leading clinical entity that usually hampers platinum-based chemotherapy, aside from nephro- and hepatotoxicity. The most frequently reported manifestations of neurotoxicity are due to the clinical onset of peripheral neurotoxicity (numbness, tingling, or paresthesia in fingers and/or toes). With prolonged treatment, they gradually lead to disturbance of proprioception, which may result in ataxic gait [3]. The clinical manifestations of encephalopathy accompanying platinum-based therapy usually appear with an increase in cumulative dose [4]. Sensory manifestations of platinum compound-induced neuropathy are often accompanied by ototoxicity (mainly hearing loss). Note that carboplatin neurotoxicity is negligible compared to cisplatin and oxaliplatin.

The root cause of many adverse effects and the organ injury of platinum-based drugs is the generation of reactive oxygen species (ROS) [5]. The increase in ROS production associated with neuronal mitochondrial dysfunction was described as a potential mechanism of platinum drug neurotoxicity. Therefore, antioxidant supplementation to reduce ROS, or to alleviate the effects of ROS, could also have a significant influence on the neurotoxic adverse effects of platinum drugs.

In conclusion, administration of oxaliplatin at therapeutic dose could induce neuropathy. Accordingly, an important therapeutic strategy is to reduce the oxaliplatin dose. However, once the therapeutic dose of oxaliplatin is reduced, the therapeutic effects can fall significantly.

Phycocyanin (PC) is one of the main pigments of the algae *Spirulina*, which also contains proteins, β-carotene, assimilable iron, γ-linoleic acid, and vitamin B12, and is used as a dietary supplement. The chemical structure of PC is very close to the structure of bilirubin, a degradation product of hemoglobin. Bilirubin is known to be an important physiological antioxidant that combats reactive oxygen species.

In 1998, Cuban researchers described the antioxidant activity of PC [6] for the first time. These authors determined that PC was able to scavenge the hydroxyl radical (OH^-^), the alkoxyl radical (RO), and the superoxide anion (O_2_^−^). It was also able to inhibit lipid peroxidation [6]. In addition, they described the anti-inflammatory properties of PC for the first time [7].

In 2018, Renugadevi et al. revealed that the PC pigment could be a promising antioxidant compound and with further study, that it has the potential as an antioxidant agent to overcome oxidant-induced diseases [8].

Our hypothesis is to test Spirulina liquid extract that has been greatly enriched with PC (SLPC) in patients with gastrointestinal cancer and treated with platinum salts, to reduce or cancel out the neuropathy associated with this treatment, which is still an unresolved medical need. The SLPC contained in the food supplement SPIRULYSAT® (produced by AlgoSource) has already been tested for metabolic syndrome (NCT02817620). Studies of SPIRULYSAT® in the murine model of non-alcoholic steatohepatitis have shown very strong anti-oxidant activity [9]. The SLPC used in this protocol will be five times more concentrated but is still produced by the same laboratory (PHYCOCARE®).

In brief, firstly, it is generally accepted that no pharmacologic agent has been definitively shown to prevent platinum salt-induced neurotoxicity [10]. Secondly, although duloxetine, a non-selective serotonin reuptake inhibitor antidepressant with non-opioid pain-relieving properties, has been shown to be effective in ameliorating neuropathic pain, clinical experience has not, to date, validated the trial data [11]. Thirdly, as already mentioned, the dose of platinum salts can be reduced so as not to induce neuropathy, possibly leading to a drastic reduction in the therapeutic effects. Fourthly, spirulina and more precisely PC has anti-oxidant effects and in murine models, it protects the kidneys from the nephrotoxic effects of cisplatin [12].

Taken together, we hypothesize that PC, and more precisely SLPC (contained in PHYCOCARE® - AlgoSource) may have protective effects against oxaliplatin-induced neuropathy in the treatment of GI cancer including esogastric, colorectal, and pancreatic cancers. Our trial will be a double-blind randomized placebo-controlled study.

### Objectives {7}

The primary objective is to demonstrate a 50% decrease in neurotoxicity grades of 2 or above at cycle 9 (4 months after the start of oxaliplatin-based chemotherapy) in the SLPC arm.

The secondary objectives are to compare the following in both arms:

- ➢ Percentage of patients who stopped oxaliplatin due to neurological toxicity
- ➢ Percentage of patients with an oxaliplatin dose decrease
- ➢ Neurological toxicities according to the Common Terminology Criteria for Adverse Events (CTCAE) v5.0
- ➢ Overall toxicity (including hematological toxicity, gastrointestinal toxicity, etc.)
- ➢ Patient’s quality of life according to the EORTC-QLQ-C30 questionnaire

### Trial design {8}

PROPERTY is a non-comparative, randomized (ratio 1:1), double-blind, multicenter placebo-controlled study. The trial was constructed like a phase 2 drug trial and is not powered for direct comparison between the two arms.

## Methods: participants, interventions, and outcomes

### Study setting {9}

This study is a French multicenter trial. Patients will be recruited from seven teaching hospital gastroenterology departments: CHU Nantes, Foch Hospital, Vendée Departmental Hospital, Estaing Hospital, Saint-Grégoire Private Hospital, Saint Nazaire Hospital, Georges and François Leclerc Center.

### Eligibility criteria {10}

The study population only includes patients with histologically or cytologically proven gastrointestinal cancer, including esogastric, colorectal, and pancreatic cancers, where the plan is to treat them with oxaliplatin. Other inclusion criteria are patients aged 18 years and over, a negative pregnancy test for women of child-bearing potential, patients with an ECOG performance status of 0 or 1 [13], and life expectancy ≥12 weeks. The results of laboratory analyses and the other inclusion criteria are shown in Table 1, as are the exclusion criteria. The main exclusion criteria are patients with known meningeal or brain metastases previously treated for their metastatic cancer or treated with oxaliplatin, and suffering from peripheral neuropathy.

**Table 1:** Inclusion and exclusion criteria

Inclusion criteria	Exclusion criteria
• Male or female with the age > or = to 18 years old. • Negative pregnancy test for women with child-bearing potential if applicable (without hysterectomy for example) • Information given to the patient who must have signed informed consent • Patient with Histologically or cytologically proven gastrointestinal adenocarcinoma including oesogastric, colorectal, and pancreatic cancers and planned to be treated with oxaliplatin • Patient with metastasic disease not previously treated • Previous radiotherapy is authorized if discontinued ≥15 days prior to randomization • Sites of disease evaluated within 42 days prior C1 day 1 of chemotherapy with thoracic-abdominal-pelvic CT scan (or abdominal-pelvic MRI and chest X-ray) • Patient with ECOG Performance status 0 or 1 • Patients with a Life expectancy ≥12 weeks • Laboratory results: ° Hematologic function: ▪ Polynuclear neutrophils ≥ 1.5.10^9^/L ▪ Platelets ≥100.10^9^/L ▪ Haemoglobin ≥9 g/dL ° Hepatic function: ▪ Transaminases ≤2.5 times upper limit of normal (ULN) (≤5 ULN in case of hepatic metastases), ▪ Alkaline phosphatases ≤2.5 × ULN (≤5 ULN in case of hepatic metastases), ▪ Total bilirubin ≤1.5 × ULN ° Renal function: ▪ Creatinemia clearance >50 ml/min (Cockcroft and Gault) • Patient with Public Health insurance coverage	• Patients with phenylketonuria • Patients with known meningeal or brain metastases • Patient previously treated for their metastatic cancer • Patient previously treated with oxaliplatin • Patient with specific contraindication or known hypersensitivity to spirulina • Patient with specific contraindication or known hypersensitivity to oxaliplatin. • Known allergy or hypersensitivity to antibodies or any preservatives if patient is treated with a monoclonal antibody combined to chemotherapy (bevacizumab or cetuximab or panitumumab or nivolumab), • Patient with clinically significant coronaries affection or myocardial infarction within 6 months prior to randomization. • Patient with peripheral neuropathy >1 (CTCAE scale version 5.0). • Patients with known dihydropyrimidine dehydrogenase (DPD) deficiency. • Patient with acute intestinal obstruction or sub-obstruction, history of inflammatory intestinal disease or extended resection of the small intestine or presence of a colic prosthesis. • Patient with unhealed wound, active oesogastric or duodenal ulcer, or bone fracture • Patient with an history of abdominal fistulas, trachea-esophageal fistulas or any other grade 4, gastro-intestinal perforations or non-gastrointestinal fistulas or intra-abdominal abscesses during the 6 months before randomization. • For patient treated with bevacizumab: patient with uncontrolled arterial hypertension (systolic pressure >150 mmHg and/or diastolic pressure >100 mmHg) with and without antihypertensive medication. Patients with high hypertension are eligible if antihypertensive medication lowers their arterial pressure to the level specified by the inclusion criterion. • Patient with an history of hypertensive crisis or hypertensive encephalopathy • Patient with other concomitant malignancy or history of cancer (except in situ carcinoma of the cervix, or non-melanoma skin cancer, treated with curative intent treatment) except if considered in complete remission for at least 2 years before randomization • Existence of any other pathology, metabolic problem, anomaly during the clinical examination or biological anomaly which may reasonable suspect an underlying pathology which would contra-indicate the use of the study medication or any other risk of complication related to the treatment. • Any treatment including an experimental drug, or participation in another clinical trial within 28 days before randomization. • Pregnant women, or women who could possibly be pregnant (or who expect to fall pregnant within 6 months of the end of treatment), or who are breast feeding are not eligible. • Men and women of child-bearing potential who do not accept to use a highly effective contraceptive (as per currently acceptable institutional standards) or abstinence during the study and for the 6 months after the last administration of the study treatments. • Persons deprived of liberty or under guardianship. • Psychological, familial, sociological or geographical condition potentially hampering compliance with the study protocol and follow-up schedule

### Who will take informed consent? {26a}

Patients’ written consent will be obtained by the investigator, their oncologist, prior to any study-specific procedures. Participation is voluntary, individuals may withdraw at any stage and participation does not affect the individual’s treatment.

### Additional consent provisions for collection and use of participant data and biological specimens {26b}

Not applicable as no biological specimens were collected as part of this trial.

## Interventions

### Explanation for the choice of comparators {6b}

Peripheral neuropathy refers to symptoms arising from damage to the peripheral nerves. These nerves carry sensation, control arm and leg movements, and control the bladder and bowel. Due to the neuropathy, the patient should avoid injury by paying attention to home safety, such as by using handrails on stairs to prevent falls and potholders in the kitchen to avoid burns. The physician can prescribe over-the-counter pain medications, lidocaine patches, menthol creams, etc. Physical therapy, occupational therapy, and rehabilitation may be helpful to regain function. In some cases, nerve damage may be permanent. The only way to reduce neurotoxicity is to decrease the dose and/or duration of oxaliplatin, which is not feasible when a long, intense dose is needed, especially when the patient has metastatic cancer. If our hypothesis is validated, it will allow us to add this dietary supplement to the patient’s follow-up to reduce neurotoxicity while continuing the chemotherapy.

Spirulina is considered a controlled foodstuff in the European Union. The species *A. maxima* and *A. platensis* are listed under the genus Spirulina (European Parliament and Council Regulation (EC) No 396/2005 of 23 February 2005 on maximum residue levels of pesticides in or on food and feed of plant and animal origin). Spirulina has been the subject of preclinical and clinical studies. Preclinical data showed no toxicity of spirulina at high doses (for administered doses up to 30 g/kg/day or ad libitum in mice). In the numerous clinical studies carried out at doses of up to 19 g/day of dry spirulina, only a few gastrointestinal and headache-type adverse effects relating to the consumption of spirulina were reported. Products containing spirulina may be contaminated with cyanotoxins, bacteria, or trace metal elements (Report of the French Agency for Food, Environmental and Occupational Health Safety (ANSES) available from: https://www.anses.fr/fr/system/files/NUT2014SA0096.pdf). For the placebo, other than rare hypersensitivity reactions to the colorant, there would be no risk given that it is currently used in food.

The quality control information for the drugs being tested, i.e. a dietary supplement and the placebo, will be provided by AlgoSource, which has an ISO 9001:2015-certified Quality Management System.

**Fig. 1:**
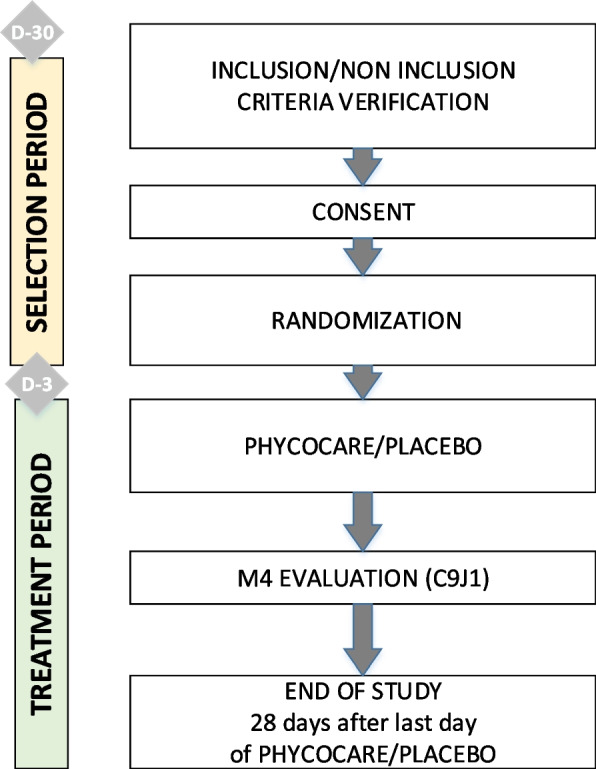
Study diagram

In relation to the primary objective, the efficacy of the SLPC will be measured at 4 months after the beginning of chemotherapy treatment, corresponding to the ninth cycle of chemotherapy. As mentioned previously, chronic toxicity does not become apparent until cycles 8–10 in the commonly used FOLFOX dosing regimen. The study diagram is shown in Fig. 1.

### Intervention description {11a}

Being double blind, the study treatment, the comparator and its corresponding placebo will be managed by the sponsor and AlgoSource. The patient must begin the study treatment 3 days before the chemotherapy regimen. This regimen is counted in cycles that last around 14 days and begin on Day1 (D1) of each chemotherapy cycle. No study treatment must be taken on chemotherapy treatment days.

**Fig. 2:**
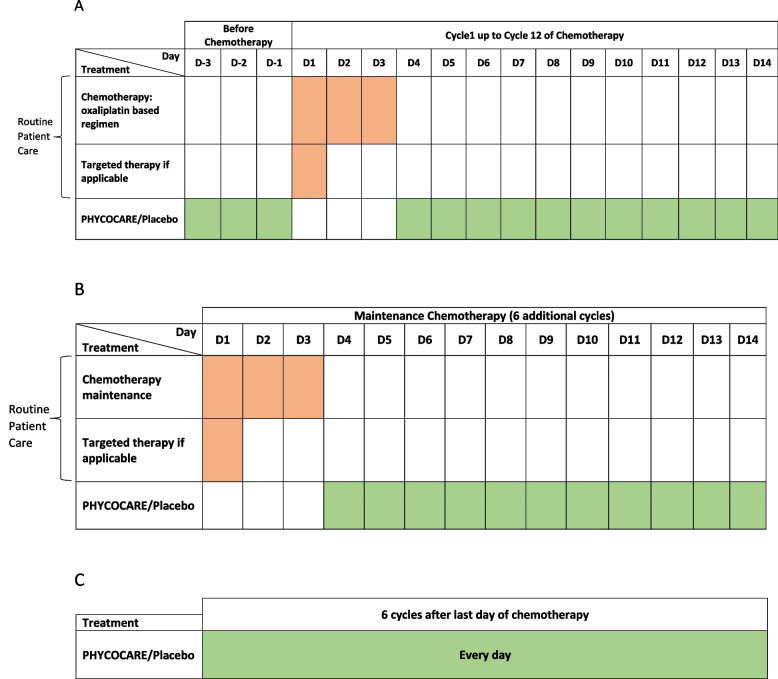
Schedule of trial treatment in chemotherapy cycles

The SPLC/placebo prescription will cover 1 month of treatment. Participants will be given a patient diary for the entirety of the study. From D3 to D1 before the first chemotherapy cycle: patients will take SPLC or placebo (one ampoule per day, neat or diluted in a glass of water). If vomiting occurs immediately after taking the SLPC dose, the patient should retake the SLPC dose. On chemotherapy days (D1 to D3), the patient does not take SLPC/placebo. From D4 to D14 SLPC/placebo must be taken once a day and 1 h before or 2 h after food and taking the antiemetic. The same schedule will be used during induction chemotherapy for 12 cycles, as shown in Fig. 2A.

After induction chemotherapy, the patients on maintenance therapy or targeted therapy will continue to take spirulina for six cycles (3 months) after the last cycle of oxaliplatin (Fig. 2B). The same schedule will be applied for the patients who are not on maintenance chemotherapy (Fig. 2C). The complete trial schedule is shown in Table 2.

**Table 2:** Study schedule

Assessments	Inclusion visit D-30 to D-4	Cycle 1	Cycle 1 to 12		Cycle 13 to 18	End of study visit / 28 days after last day of PHYCOCARE® /PLACEBO
		D-3 to D-1	D1 to D3	D4 to D14	D1 to D14	
Informed consent	X					
Medical History	X					
Clinical examination (Performance status (ECOG), vital signs, symptoms)	X		X (D1)		every 28 days	X
ONLS questionnaire	X		at M4 (C9D1)			X
QLQ-C30 questionnaire	X		at M4 (C9D1)			X
EDX	X		at M4 (C9D1)			X
Randomisation	X					
Scanner	at D-42					
PHYCOCARE® or placebo treatment		X		X	X (days without chemotherapy)	
Oxaliplatin-based chemotherapy			X			
Chemotherapy maintenance regimen					If applicable	
Adverse events (AE)		AE assessed at each patient visit				X
Treatment adherence (patient diary)		Compliance assessed at each patient visit				X

Note that the oxaliplatin dose is 85 mg/m^2^ and the dose adjustment is shown in Table 3.

**Table 3:** Oxaliplatin dose adjustement

Scale	NCI-CTC grading for neuropathy
Grade 1	Asymptomatic, loss of deep tendon, reflexes, or paresthesia (including tingling), but not interfering with function
Grade 2	Sensory alteration or paresthesia (including tingling) interfering with function, but not ADL
Grade 3	Sensory alteration or paresthesia interfering with ADL
Grade 4	Disabling

### Criteria for discontinuing or modifying allocated interventions {11b}

The study treatment can be discontinued early at any time: at the request of the patient, whatever the reason; at the request of the investigator for any reason in keeping with the patient’s best interests; at the request of the sponsor; in case of non-confirmation of the selection criteria; and in case of unacceptable toxicity.

### Strategies to improve adherence to interventions {11c}

Patient adherence to treatment will be assessed at each visit and at the end of the study through the patient diary.

### Relevant concomitant care permitted or prohibited during the trial {11d}

Any drug deemed necessary for the patient’s well-being may be administered by the investigator. However, patients are not permitted to take plant-based therapy (gemmotherapy or phytotherapy).

### Provisions for post-trial care {30}

At the end of the clinical research, the patient will be followed up by their oncologist and will benefit from the usual care for their disease.

The sponsor has taken out an insurance policy covering the financial consequences of its civil liability in compliance with the regulations.

### Outcomes {12}

The primary endpoint is the rate of neurotoxicity according to US NCI (National Cancer Institute) criteria in both arms 4 months after the beginning of oxaliplatin-based regimen. NCI criteria are listed in Table 4.

**Table 4:** US National Cancer Institute common toxicity criteria (NCI-CTC)

Oxaliplatine dosage adjustment according to neurotoxicity				
Neurotoxicities	Grade	Toxicity duration		
		1 to 7 days	> 7 days	Persistent between cycles^**a**^
Paresthesias/dysesthesias^b^ that do not interfere with fine motor skills	1	No dose reduction		
Paresthesias/dysesthesias^b^ that interfere with fine motor skills but do not affect activities of daily living (ADLs)	2	No dose reduction		75% of the dose
Paresthesias/dysesthesias^b^ with pain or decreased fine motor skills that affect activities of daily living (ADLs)	3	1st time: 75% of the dose 2nd time: 50% of the dose		Discontinue treatment
Persistent or incapacitating or lifethreatening paresthesia/dysesthesia	4	Discontinue treatment		
Acute toxicity: laryngeal-pharyngeal dysesthesia^b^ (during or within 2 h after infusion)	NA	Extend the oxaliplatin infusion to 6 h on the next treatment.	NA	

^a^Toxicity still present at the beginning of the next cycle

^b^May be caused by the cold

The secondary outcomes are:

- ⇨ Time frame for definitive discontinuation or decrease in oxaliplatin treatment. The dose or discontinuation of oxaliplatin will be noted at each chemotherapy visit, in accordance with the process established in Table 3
- ⇨ Dose intensity of oxaliplatin noted at each chemotherapy visit (see Table 3)
- ⇨ Neurological adverse events at the following hospital visits: baseline, M4, and M9/termination visit
- ⇨ Nerve electrodiagnostic examination (EDX) and neurology questionnaire: Overall Neuropathy Limitation Scale (ONLS) French translation according to Graham et Hugues [14] at baseline, M4 and M9/termination visit
- ⇨ Adverse Events collected at each chemotherapy visit or in patient diary
- ⇨ Quality of life questionnaire: QLQ C30 at baseline, M4 and M9/termination visit

Note that when a patient discontinues the study treatment early for whatever reason, an early termination visit will be conducted if the patient’s condition allows.

### Participant timeline {13}

The participants’ treatment period corresponds to 9 months (18 cycles) and the participation period to 11 months.

### Sample size {14}

Simon’s two-stage design [15] will be used. The null hypothesis that the true response rate is 0.60, the proportion of neurotoxicity targeted, will be tested against a one-sided alternative. In the first stage, 31 patients will be accrued. If there are 18 patients or fewer with grade 1 neurotoxicity among these 31 patients in the SLPC arm, the study will be halted. Otherwise, 24 additional patients will be accrued, making a total of 55. The null hypothesis will be rejected if 38 or more responses are observed in 55 of the SLPC arm patients. With a type I error rate of 0.05 and a power of 0.90, 55 patients will be enrolled in each arm, that is to say, 110 patients.

All the previous hypotheses account for a lost-to-follow-up rate at 4 months of 20%.

### Recruitment {15}

Recruitment is planned over a period of 24 months. The rate of one patient per center per 2 months makes this recruitment target completely achievable.

## Assignment of interventions: allocation

### Sequence generation {16a}

Randomization will be conducted blindly by center, neuropathy (stage 0 or 1), and age (< 65 years old and ≥ 65 years old). Wrongly enrolled randomized patients will not be replaced. It will be block balanced in a 1:1 ratio. The software used for the randomization is SAS version 9.4.

### Concealment mechanisms {16b}

Randomization will be carried out using EnnovClinical software, *the e-CRF of CHU Nantes*, by connecting to the website at https://nantes-lrsy.ennov.com/EnnovClinical/login. The connection will be made using a login, a password, and a study number, issued by a data manager from the Sponsor Department. The enrolment number and randomization arm will be assigned automatically. Subjects are randomized into blocks as the allocation progresses. A block, *generated as already said by SAS*, being a subgroup of a predetermined size within which there is a random allocation of patients.

### Implementation {16c}

The randomization key is known only to the biostatistician and the data managers, to make it impossible for the investigator to assign a particular treatment. At the randomization visit, which can take place at the enrolment visit, the investigator will check the inclusion and *exclusion* criteria and then validate the randomization on the electronic Case Report Form (eCRF).

## Assignment of interventions: blinding

### Who will be blinded? {17a}

Our study is a randomized double-blind trial. Randomization will take place at the enrolment visit between D30 and D4. The patients and the investigators and their teams will be blind and will not know which treatment has been assigned.

### Procedure for unblinding if needed {17b}

Unnecessary or unintended decoding of the blinding should not occur during the study. If unblinding is deemed to be necessary for reasons of emergency, the investigator should send the completed “Unblinding Request Form” to the sponsor by email or by fax. The Coordination Committee will review the request. The coordinating pharmacy at CHU Nantes will have access to the correlation between the randomization number and the allocation for that person. If the request is approved, the results of any unblinding procedure will only be presented to the local Principal Investigator and the team treating the patient. In the case of a SUSAR, the vigilance team will unblind the treatment allocation for the regulatory process.

## Data collection and management

### Plans for assessment and collection of outcomes {18a}

Motor nerve conduction will be investigated for the median, tibial and fibular nerves, evaluating standard parameters: compound muscle action potential distal latency, baseline-to-negative peak amplitude and area, and motor nerve conduction velocity. Sensory nerve conduction will be recorded for the median, radial, and sural nerves, with sensory action potential peak-to-peak measurement. This EDX protocol will be performed three times for each patient, at the enrolment visit, at M4 (i.e., D1 of the ninth chemotherapy cycle), and at the end of the trial, 28 days after the last dose of SLPC/placebo.

Furthermore, we paired the ONLS questionnaire with the EDX. The ONLS scale is a disability questionnaire with a score that ranges from 0 (no disability) to 12 (severe disability). It has been validated in 100 patients with inflammatory polyneuropathy. It has a strong correlation (r = 0.97) with the Overall Disability Sum Score [14] and is widely used by neurologists assessing peripheral neuropathy.

Patients will complete another questionnaire during the trial, to measure their quality of life: QLQ-C30 version three [16]. Comprising 30 different items (questions), it is made up of 8 multi-item functional (physical, role, emotional, cognitive, and social) and symptom (fatigue, pain, and nausea) scales, one global health status and quality of life (QOL) scale, and 6 single items (dyspnea, insomnia, appetite loss, constipation, diarrhea, and financial difficulties). It covers the majority of the core symptoms recommended for patient-reported outcome measurement in cancer clinical trials [17].

### Plan to promote participant retention and complete follow-up {18b}

The target population of the trial is cancer patients during chemotherapy treatment; missing data could only result from withdrawal of consent or a serious adverse event (SAE). There is no plan for promoting participant retention.

### Data management {19}

An eCRF will be drawn up for each subject. Subjects will be identified using a code which should be the only information featuring in the eCRF, enabling a retrospective link with the patient.

The investigator will also encode the patient data in any documents that may be in their possession (EDX, questionnaires) and attached to the eCRF. At the end of the study, the CRF database and the safety database will be reconciled before the databases are locked. Similarly, an annual reconciliation will be carried out when updating the Annual Safety Report.

### Confidentiality {27}

Each patient's medical data will only be provided to the sponsor or any person duly authorized by the sponsor and, where applicable, to authorized health authorities, under conditions of confidentiality. The sponsor and the supervisory authorities may request direct access to medical records for the purposes of verification of the procedures and/or data in respect of the clinical trial, within the limits authorized by legislation and regulations. The data compiled during the trial may be processed electronically in compliance with CNIL requirements. CNIL is an independent administrative regulatory body whose mission is to ensure that data privacy legislation is applied to the collection, storage, and use of personal data.

### Plans for collection, laboratory evaluation, and storage of biological specimens for genetic or molecular analysis in this trial/future use {33}

Not applicable as no biological specimens were collected as part of this trial.

## Statistical methods

### Statistical methods for primary and secondary outcomes {20a}

Descriptive analyses will be conducted for all variables collected. Point estimates and 95% confidence intervals will be calculated for qualitative and quantitative variables. For estimates obtained using a small sample, the medians and interquartile intervals (for quantitative variables) and exact confidence interval (for qualitative variables) will be provided. All analyses will be performed with the use of R software (version 4.0) or SAS software (version 9.4).

To fulfill the main objective of this study, the prevalence of neurotoxicity of grade 2 or above in both arms will be estimated using linear mixed regression, taking into account the center effect (random effect). The second step will be to adjust this estimate based on known risk factors (gender, age, etc.).

For the secondary analyses:

- Comparison of time before cessation or reduction of chemotherapy treatment will be estimated using the Kaplan-Meier method and tested with the log-rank test;
- Comparison of patients with a decrease in treatment due to neurological toxicity will take place via mixed linear regression on both treatment arms;
- Progression of grades 3 and 4 neurological adverse events at baseline, M4, and M9 (or at the last visit if earlier) will be tested using mixed linear regression with a random center effect;
- EDX data will be compared using Fisher’s exact test and the Wilcoxon-Mann-Whitney test;
- The chi-square test or Fisher’s exact test will be used to compare Adverse Events in both arms;
- Progression of grades 3 and 4 neurological QLQ C30 at baseline, M4, and M9 (or at the last visit if earlier) will be tested via mixed linear regression with a random center effect; and
- Median progression-free survival will be estimated and plotted using the Kaplan-Meier method and the response rate will be estimated as a percentage with a 95% confidence interval.

### Interim analyses {21b}

A futility analysis will be performed according to Simon’s method [15]. Enrolment will continue during the futility analysis. The study will be halted if there are fewer than 18 responders (i.e., patients with grade 1 neurotoxicity) in the first 31 patients enrolled in the SLPC arm.

### Methods for additional analyses (e.g., subgroup analyses) {20b}

No additional analyses are planned.

### Methods in analysis to handle protocol non-adherence and any statistical methods to handle missing data {20c}

If missing, unused, or invalid data are highlighted, two methods will be used. With regard to the prevalence rates detailed in the objectives, estimates will be created based on the modified intention-to-treat population. For the primary objective only, multiple imputation techniques will be applied to all the covariates and to neurotoxicity grade for sensitivity analyses.

### Plans to give access to the full protocol, participant-level data and statistical code {31c}

The datasets analyzed in the present study and the statistical code are available from the corresponding author on reasonable request, as is the full protocol.

## Oversight and monitoring

### Composition of the coordinating center and the trial steering committee {5d}

The Scientific Committee was created and coordinated by Prof. J. Bennouna and Dr C. Le Gouill. Its membership comprises Prof Y. Péréon, neurologist, a biostatistician and methodologist, the study coordinator, and the project manager of the clinical investigation center (CIC 1413). The Steering Committee is composed of the members of the Scientific Committee with the addition of the data management team, the study nurse who coordinates assistance for patient enrolment in the other centers, and the monitoring Clinical Research Assistant. The sponsor’s project manager coordinates this committee and drafts the “Property newsletter” which will include, among other things, the latest news on patient enrolments and amendments to the protocol.

### Composition of Data Monitoring Committee, its role and reporting structure {21a}

SLPC is contained in PHYCOCARE®, a dietary supplement, leading to a low-risk intervention in this study; a Data and Safety Monitoring Committee was thus irrelevant.

### Adverse event reporting and harms {22}

All subjects will be followed up for adverse event collection, through self-reporting in the patient’s diary and monthly, at the time of the visit, based on clinical and biological examination. Neurological toxicity will also be monitored by EDX. In addition, between visits, as well as all subjects receiving chemotherapy, patients are asked to contact the clinical team in case of difficulties or toxicity.

The expected ARs are associated with:

- ▪ PHYCOCARE®
  Spirulina has been the subject of preclinical and clinical studies. Preclinical data have not shown toxicity of spirulina at high doses (for administered doses up to 30g/kg/day or ad libitum in mice). In the numerous clinical studies carried out at doses of up to 19g/day of dry spirulina, only a few gastrointestinal (moderate vomiting and diarrhea episode) and headache-type adverse effects linked to the consumption of spirulina have been reported.
  Products containing spirulina may be contaminated with cyanotoxins, with bacteria or by metallic trace elements (Report of the French Agency for Food, Environmental and Occupational Health Safety (ANSES) available from: https://www.anses.fr/fr/system/files/NUT2014SA0096.pdf); no clinical impact (infection/signs of poisoning) is expected, however.
- ▪ The placebo:
  - ° Hypersensitivity reaction to colorant (dry blue) or *Cinnamomum camphora cineol* may be expected
  - ° Toxicity: camphor laurel is mildly toxic to humans, and mild symptoms may occur if large quantities are eaten. All parts of the plant are poisonous and can cause nausea, vomiting, and respiratory distress. Allergic skin reactions can also occur
  - ° Headache (moderate and intermittent),
  - ° Constipation or loose stools
  - ° Elevated transaminases

- The protocol:
  - ° Disagreement or anxiety about randomization to the placebo arm
  - ° EDX: mild discomfort related to electrical stimulation and muscle needle examination
  - ° Asthenia, boredom, due to difficulty or inability to complete the questionnaires,
- The disease:
  - ° Anxiety/depression (including sleep disorders, suicide attempts)
  - ° Progression of the disease
  - ° Metastasis and related symptoms
- Chemotherapy and associated treatments. The main adverse reactions due to chemotherapy are:
  - ° Hematological toxicity
  - ° Gastrointestinal toxicity (nausea, vomiting, diarrhea, stomatitis)
  - ° Cutaneous-mucosal toxicity
  - ° Neurological toxicity
  - ° Fatigue
  Chemotherapy also requires standard prophylaxis for drug toxicity and the risk of thrombosis, and pain killers.

All these drugs are used in their marketing authorization indications, so the expected adverse drug reactions are as listed in each Summary of Product Characteristics.

### Frequency of and plans for trial conduct auditing {23}

An inspection or audit may take place as part of this study, performed by the sponsor and/or by the regulatory authorities. Inspectors will check the documents, logistics, records, and any other resources that the authorities consider to be associated with the clinical trial and that may be located at the trial site itself.

### Plans for communicating important protocol amendments to relevant parties (e.g. trial participants, ethical committees) {25}

The amended protocol will be a dated, updated version. If necessary, the information form and consent form should be amended. The sponsor project manager will notify the centers and a copy of the revised protocol will be sent to all the principal investigators to add to the Investigator Site File. The updated protocol is currently at version 2 as at May 23, 2022. All the submissions/declarations were made by the Sponsor Department at CHU Nantes to the French regulatory authority (ANSM) and the Ethics Committee (*Comité de Protection des Personnes Ile de France VIII – Nantes*). The Sponsor Department’s clinical research associate will report any deviations from the protocol, which will be fully documented using a breach report form.

### Dissemination plans {31a}

The trial results will be published in international oncology, medical and scientific journals and presented at national and international conferences.

## Discussion

Although oxaliplatin has an improved overall survival rate [18, 19], oxaliplatin-induced peripheral neuropathy (OIPN) remains a treatment-limiting factor [20]. Some degree of OIPN occurs in nearly all patients [18] and approximately two thirds will have symptoms 1 year post treatment or beyond [21]. OIPN has been reported as dose dependent, with symptoms more likely to occur as the cumulative dose exceeds 780–850mg/m^2^. Unlike acute OIPN, which is transient, chronic OIPN can persist for months or years [22] and includes pain, numbness, and dysesthesias as well as gait disturbances that lead to reduced quality of life and function [23].

The US National Cancer Institute’s Symptom Management and Health-Related Quality of Life Steering Committee has announced that CIPN is a priority area of translational research in cancer care [24]. Even the latest literature data clearly indicate that although numerous preventative therapies were tested for their potential utility for CIPN alleviation, this clinical entity currently is still not preventable, and many strategies tested were found to be ineffective [25, 26].

At present, based on meta-analyses of clinical trials, no drug can be proposed as a gold standard to either prevent CIPN or treat its symptoms, and chemotherapeutic drug dose modification remains the only preventative strategy [27].

Our trial will provide a significant clinical gain for the patient if our hypothesis is correct. The oncologist will be able to continue the same treatment at the same dose with a therapeutic response to neuropathy.

## Trial status

This trial has just started.

The updated protocol is at version 2 as of May 23, 2022.

The first patient was enrolled in April 2022.

Recruitment by the investigating centers is planned to continue until April 2024.

## Supplementary Information

**Additional file 1.** PROPERTY’s Informed consent.

## Data Availability

Data collected during the test may be processed electronically, in accordance with the requirements of CNIL (compliance with reference methodology MR001). The investigators will share the entirety of the final trial dataset.

## Abbreviations

ADL: Activities of daily living
CIPN: Chemotherapy-induced peripheral neuropathy
CNIL: French Data Protection Authority
CMAP: Compound muscle action potential
D: Day
eCRF: eCase Report Form
EDX: Electrodiagnostic examination
GI: Gastrointestinal
M: Month
NCI-CTC: US National Cancer Institute common toxicity criteria
OIPN: Oxaliplatin-induced peripheral neuropathy
ONLS: Overall Neuropathy Limitation Scale
PC: Phycocyanin
QOL: Quality of life
ROS: Reactive oxygen species
SCV: Sensory conduction velocities
SLPC: Spirulina liquid extract greatly enriched in PC
