# Supplementary material for: PROPERTY: study protocol for a randomized, double-blind, multicenter placebo-controlled trial assessing neurotoxicity in patients with metastatic gastrointestinal cancer taking PHYCOCARE® during oxaliplatin-based chemotherapy
Source: Trials. 2023 Jan 20;24:50. doi: 10.1186/s13063-023-07071-z (PMC9854012; doi:10.1186/s13063-023-07071-z)
Supplement: Supplementary file 1 — Additional file 1. PROPERTY’s Informed consent. [file 13063_2023_7071_MOESM1_ESM.pdf]

CENTRE HOSPITALIER UNIVERSITAIRE DE NANTES

Note d'information pour la participation à la recherche

« Étude randomisée, en double aveugle, multicentrique*, contrôlée par PLACEBO*, évaluant la neurotoxicité* chez des patients atteints d'un cancer gastro-intestinal métastatique prenant du PHYCOCARE ou un PLACEBO pendant une chimiothérapie* à base d'oxaliplatine »

Titre abrégé : « PROPERTY»

Promoteur : CHU de Nantes

N° Enregistrement : 2021-A01568-33

SOMMAIRE

SOMMAIRE 1
GLOSSAIRE DES TERMES TECHNIQUES 2
INFORMATION SUR LE PRODUIT A L'ETUDE 3
BUT DE LA RECHERCHE 3
DEROULEMENT DE LA RECHERCHE..................... 3
RISQUES DE LA RECHERCHE 5
BENEFICES ATTENDUS DE LA RECHERCHE 6
ALTERNATIVES A LA RECHERCHE 6
FIN DE LA RECHERCHE 6
FRAIS SUPPLEMENTAIRES 6
VOS DROITS PENDANT LA RECHERCHE 6
LE CADRE REGLEMENTAIRE 8
EN RESUME… 8
ANNEXE 1 : CALENDRIER DES VISITES DE LA RECHERCHE 10

VOS CONTACTS :

Pour toute question concernant l'étude, retrait de consentement, ou pour exercer vos droits concernant vos données (accès, rectification, etc…), votre contact privilégié est :

L'investigateur coordonnateur de la recherche :

Dr LE GOUILL-JAIJARAT Christèle

✉ Service de Hôpital de jour d'oncologie médicale (unité pluridisciplinaire)- CHU de Nantes

☎ 02.40.08.32.81 / christele.legouill@chu-nantes.fr

Pour toute question générale sur le traitement de vos données :

Le promoteur de la recherche, responsable du traitement des données personnelles :

CHU de Nantes, direction de la recherche et de l'innovation

5 allée de l'Ile Gloriette, 44093 NANTES Cedex 1

Le Délégué à la Protection des Données (DPD - Data Protection Officer (DPO)) :

vosdonneespersonnelles@chu-nantes.fr

GLOSSAIRE DESTERMESTECHNIQUES

- Chimiothérapie : Une chimiothérapie est un traitement général qui vise à détruire les cellules cancéreuses ou à les empêcher de se multiplier dans l'ensemble du corps. Elle peut être administrée par perfusion, piqûre ou sous forme de comprimés.

- Chimiothérapie à base d'oxaliplatine : les chimiothérapies à base d'oxaliplatine sont des associations de traitements dont un des traitements est l'oxaliplatine

- Double aveugle : cela signifie que ni vous ni l'investigateur* n'auront connaissance du traitement qui vous sera administré. En effet, les résultats du tirage au sort ne seront connus que du pharmacien qui délivrera le traitement. Cette méthode permettra à l'investigateur d'être le plus objectif concernant les effets des traitements.

- Electromyogramme : c'est un examen appelé exploration fonctionnelle, qui permet d'évaluer le fonctionnement des nerfs et des muscles. Il consiste à appliquer un faible courant électrique au niveau des fibres nerveuses, sensitives ou motrices, du système nerveux. Pour cela, des électrodes (petits patchs) sont posées sur la peau et sont reliées chacune à l'appareil de stimulation (l'électromyogramme ou EMG). Un faible courant électrique est envoyé dans les électrodes qui vont alors diffuser ce courant jusqu'aux muscles qui vont se contracter et bouger. Les électrodes vont enregistrer en retour l'activité des muscles et la transmettre à l'appareil. L'appareil va analyser l'activité et la reproduire sous forme de tracés.

- Gemmothérapie : utilisations de bourgeons de plantes ou de médicaments à base de bourgeons de plantes ((poudres, préparations en ampoules, infusions…) pour soigner les maux du corps. La gemmothérapie est une branche de la phytothérapie.
- Médecin coordonnateur : Médecin spécialiste de recherche clinique qui dirige, surveille et coordonne la réalisation de l'essai clinique à l'échelle de tous les centres participants
- Médecin investigateur : Médecin spécialiste de recherche clinique qui dirige et surveille la réalisation de l'essai clinique dans le centre qui vous suit. Pour vous, ce sera votre médecin oncologue
- Multicentrique : se dit d'un projet qui se déroule dans plusieurs centres
- Neuropathie : affection du système nerveux contrôlant les nerfs moteurs et sensitifs des membres et les nerfs contrôlant le système nerveux autonome qui commande les organes. Les neuropathies peuvent se présenter par exemple sous la forme de fourmillements (atteintes des nerfs sensitifs), de faiblesse dans les muscles (atteinte des nerfs moteurs) ou de constipations (atteinte des nerfs autonomes)

- Neurotoxicité : l'action d'un traitement ou d'une substance sur le système nerveux. Dans cette étude, l'oxaliplatine a une action neurotoxique possible chez certains patients (exemple d'effets : difficultés à réaliser des gestes précis comme fermer un bouton de chemise, fourmillements, modification du goût, étourdissements, maux de tête). Cette toxicité est prise en charge par des traitements et par la réduction de la dose d'oxaliplatine ou l'arrêt de l'oxaliplatine.

- Phytothérapie : utilisation de plantes ou de médicaments à base de plantes (poudres, préparations en ampoules, infusions…) pour soigner les maux du corps

- PLACEBO : médicament sans principe dit « actif », sans effet sur le corps.

- Promoteur : Le promoteur est la personne physique, la société ou l'institution qui prend l'initiative d'un essai clinique et en assume les responsabilités et le financement.

- Randomisation : Les participants sont répartis par tirage au sort (par un logiciel informatisé) dans les deux groupes de traitement de façon à assurer une comparaison équitable des deux traitements.

- Recherche interventionnelle : se dit d'une recherche/étude portant sur la personne humaine, qui comporte un acte réalisé en plus de la prise en charge habituelle. Cet acte peut être par exemple, une stratégie thérapeutique, un examen diagnostic ou de surveillance de la maladie étudiée.

Madame, Monsieur,

Votre médecin vous a informé que vous êtes atteint(e) d'un cancer gastro-intestinal métastatique et vous propose de participer à une recherche interventionnelle* nommée PROPERTY « Étude randomisée, en double aveugle*, multicentrique*, contrôlée par PLACEBO*, évaluant la neurotoxicité* chez des patients atteints d'un cancer gastro-intestinal métastatique prenant du PHYCOCARE ou un PLACEBO pendant une chimiothérapie* à base d'oxaliplatine ».

Cette recherche qui vous est proposée a pour but de tester l'effet protecteur du PHYCOCARE sur la neuropathie* induite par l'oxaliplatine.

Le CHU de Nantes est le promoteur* de cette recherche, c'est à dire qu'il en est responsable et qu'il l'organise.

Votre participation à la recherche, au cas où vous donneriez votre accord, ne pourra vous être confirmée qu'à la condition que vous remplissiez tous les critères d'inclusion pour participer à cette recherche.

- Votre médecin, c'est-à-dire l'investigateur de l'étude, vous a donné des explications. Elles sont résumées dans ce document intitulé « note d'information ». Nous vous invitons à le lire attentivement avant de vous décider. Vous avez un délai de réflexion avant de vous décider lors de la visite d'inclusion.
- Si vous décidez de participer à cette recherche, nous vous demanderons de dater et signer une attestation de consentement. Cette attestation sera aussi signée par l'investigateur. Cette signature confirmera que vous êtes d'accord pour participer à la recherche. Votre signature est indispensable, ainsi que celle de l'investigateur. Même après avoir signé pour donner votre accord de participation, vous garderez le droit d'interrompre à tout moment votre participation sans avoir à vous justifier et sans préjudice sur votre prise en charge future.
- Tous les termes techniques seront suivis d'un astérisque (*) et seront définis dans le glossaire au début de ce document d'information.

INFORMATION SUR LE PRODUIT AL'ETUDE

Le PHYCOCARE contient de la phycocyanine, une molécule extraite d'une micro algue. Elle est reconnue comme un aliment par l'Organisation des Nations Unies pour l'Alimentation et l'Agriculture. En France, la phycocyanine est employée à des fins alimentaires, notamment sous la forme de compléments, en raison de son potentiel nutritif, ou comme colorant.

BUT DELARECHERCHE

Nous émettons l'hypothèse que le PHYCOCARE pourrait protéger des effets indésirables neurologiques* induits par l'oxaliplatine dans le traitement des cancers gastro-intestinaux métastatiques.

Pour vérifier cette hypothèse, deux groupes de patients seront inclus : un groupe qui recevra le PHYCOCARE et un autre groupe qui recevra le PLACEBO*.

Le but de cette recherche est de comparer la neurotoxicité* des patients prenant du PHYCOCARE des patients prenant un PLACEBO pendant une chimiothérapie* à base d'oxaliplatine, Ce traitement de chimiothérapie (oxaliplatine) est usuellement prescrit dans le cadre du traitement de votre maladie et peut provoquer des atteintes des nerfs appelées neuropathies*.

DEROULEMENT DE LARECHERCHE

Si vous acceptez de participer, un tirage au sort, appelé aussi randomisation*, aura lieu lors de votre inclusion afin de déterminer le groupe de traitement : soit le PHYCOCARE soit le PLACEBO*. Environ 110 patients inclus seront inclus dans cette recherche avec une répartition équivalente entre les groupes.

Cette recherche se déroule en double aveugle*, cela signifie que ni vous ni l'investigateur* n'auront connaissance du traitement qui vous sera administré. En effet, les résultats du tirage au sort ne seront connus

que du pharmacien hospitalier qui délivrera le traitement. Cette méthode permettra à l'investigateur d'être le plus objectif concernant les effets des traitements.

Votre participation éventuelle à cette recherche durera maximum 11 mois.

Votre prise en charge dans le cadre de la recherche nécessitera de réaliser les visites décrites ci-après. Pendant votre période de traitement par chimiothérapie, la durée de votre présence à l'hôpital ne sera pas allongée par la recherche car ces visites font parties de votre prise en charge.
Les questionnaires et l'électromyogramme réalisés à l'inclusion et à la fin de la recherche, nécessiteront de rester environ 30 à 45 minutes de plus que pour une consultation standard.

- ***La visite de sélection (entre 30 et 4 jours avant l'attribution du groupe de traitement) :*** Celle-ci commence obligatoirement par l'explication de l'étude par votre médecin-investigateur et par le recueil de votre consentement. Aucun acte entrant dans la réalisation de l'étude ne pourra être effectué avant la signature de l'attestation de consentement. Lors de cette période, et plus particulièrement lors de la visite d'inclusion, votre médecin-investigateur vérifiera que vous ne présentez aucune contre-indication pour participer à l'étude.

  Après l'explication de l'étude et la signature du consentement, l'investigateur fera un examen clinique. Il vous demandera de répondre à deux questionnaires (le questionnaire ONLS sur les neuropathies et le questionnaire EORTC-QLQ C30 sur la qualité de vie) et de faire un électromyogramme*.

  Si vous êtes une femme, vous ne devez pas être enceinte ni en cours d'allaitement, et si vous êtes en âge de procréer, vous devez utiliser un moyen contraceptif efficace tel que la pilule. Si vous êtes un homme, vous devez également utiliser un moyen contraceptif efficace.

  La randomisation peut être faite en même temps que la visite d'inclusion. Le tirage au sort sera fait par logiciel informatisé pour savoir si vous allez avoir le PHYCOCARE ou le PLACEBO*.

- ***La période de traitement par PHYCOCARE/PLACEBO :*** Le traitement par PHYCOCARE/PLACEBO sera à prendre pendant un maximum de 18 cycles (9 mois) dont 6 cycles (3 mois) après arrêt de l'oxaliplatine. : le contenu d'1 ampoule en verre à prendre par voie orale, soit une heure avant le repas ou soit deux heures après le repas. Les ampoules pleines doivent être gardées dans l'emballage d'origine à l'abri de la lumière et à une température comprise entre 4 et 25°C.

  - J-3 à J-1 : Le traitement sera débuté 3 jours avant la première dose de chimiothérapie* à base d'oxaliplatine : une ampoule par jour de PHYCOCARE/PLACEBO pendant 3 jours

  - Pendant la chimiothérapie* à base d'Oxaliplatine : maximum 12 cycles (6mois) de PHYCOCARE/PLACEBO
    - o Les jours de chimiothérapie* : aucune prise de PHYCOCARE/PLACEBO
    - o Les jours sans chimiothérapie* : une ampoule de PHYCOCARE/PLACEBO par jour

  - Après 9 cycles (environ 4 mois) de PHYCOCARE/PLACEBO : Il vous sera demander de répondre à deux questionnaires (ONLS et EORTC-QLQ C30) et de faire un électromyogramme*.

  - Après arrêt de l'oxaliplatine :
    - o Le médecin peut décider à tout moment pendant l'étude d'arrêter l'oxaliplatine et d'adapter la chimiothérapie* :
      - Soit poursuite de l'étude avec la chimiothérapie* (sans oxaliplatine) : dans ce cas
        - Les jours de chimiothérapie* : aucune prise de PHYCOCARE/PLACEBO
        - Les jours sans chimiothérapie* : une ampoule de PHYCOCARE/PLACEBO par jour pendant 6 cycles (3 mois)
      - Soit poursuite de l'étude sans chimiothérapie* : une ampoule de PHYCOCARE/PLACEBO par jour pendant 6 cycles (3 mois) de 14 jours
      - Soit arrêt de l'étude : dans ce cas une visite de fin d'étude sera programmée 28 jours après la dernière dose de PHYCOCARE/PLACEBO

- ***La Visite de Fin d'étude (28 jours après la dernière dose de PHYCOCARE/PLACEBO)*** : Lors de cette visite, l'investigateur fera un examen clinique. Il vous demandera de répondre à deux questionnaires (ONLS et EORTC-QLQ C30) et de faire un électromyogramme*.

Pour pouvoir participer à cette recherche vous devez être affilié(e) ou bénéficier d'un régime de sécurité sociale.

Donner votre accord pour participer à cette étude impliquera que vous vous engagiez pendant toute la durée de l'étude à :

- ❖ Remplir le carnet de prise de traitement PHYCOCARE/PLACEBO à votre domicile pour le suivi de l'adhésion au traitement et le rapporter à chaque visite
- ❖ Ne prendre aucun traitement complémentaire à base de plantes (incluant la phytothérapie* et la gemmothérapie*)
- ❖ Informer l'investigateur de tous les médicaments que vous prenez.
- ❖ Répondre aux questionnaires qualité de vie et sur les neuropathies* aux visites suivantes : inclusion après 9 cycles (environ 4 mois) de traitement et la visite de fin d 'étude
- ❖ Réaliser les électromyogrammes* prévus aux visites suivantes : inclusion, après 9 cycles (environ 4 mois) de traitement et la visite de fin d 'étude
- ❖ Vous rendre à toutes les visites prévues dans le protocole : un tableau résumant les examens et visites réalisés au cours du protocole est décrit en annexe 1 du présent document.
- ❖ Informer immédiatement de tout évènement ou effet indésirable éventuellement rencontré au cours de votre participation à la recherche et reportez les événements indésirables dans votre carnet patient
- ❖ Vous ne pourrez participer à aucune autre recherche interventionnelle pendant toute la durée de votre participation à cette étude et jusqu'à 28 jours après la dernière dose de PHYCOCARE/PLACEBO, à l'exception d'études dites « non interventionnelles », ne modifiant pas le suivi habituel.

RISQUES DELARECHERCHE

Pour toute recherche interventionnelle, les traitements et les autres examens réalisés peuvent comporter des risques dont certains sont déjà connus et d'autres pas encore décrits. Vous devez savoir que les traitements auxquels vous serez soumis(e) pendant cette recherche comportent des risques et inconvénients connus.

Les risques supplémentaires par rapport à une prise en charge en dehors de cette recherche portent sur :

- La prise de PHYCOCARE/PLACEBO
- La réalisation d'un électromyogramme* à l'inclusion, après 9 cycles (environ 4 mois) et à la fin de l'étude

Risques associés à la prise de PHYCOCARE/PLACEBO : Les données précliniques n'ont montré aucune toxicité de la phycocyanine à fortes doses. Parmi les nombreuses études cliniques, seuls quelques effets indésirables de type digestif (vomissements, diarrhées) et maux de tête liés à la consommation de phycocyanine ont été signalés.

Risques associés à la réalisation d'électromyogrammes* : La durée de l'examen dépend du nombre de muscles explorés au repos et/ou après stimulation. Il dure généralement entre 30 minutes et environ une heure. Les effets indésirables connus sont : une légère gêne liée aux chocs électriques et à l'examen des aiguilles musculaires.

Le questionnaire ONLS sur les neuropathies* sera complété lors d'une visite avec le médecin investigateur. Il comporte une dizaine de questions qui vous seront posées par le médecin. Il ne présente aucun risque.

En cas de problème, et à tout moment dans la recherche, vous devrez contacter l'*investigateur :* _

Dr/PR :……………………………..Joignable au :

Email : ……………………………………………….

(Adresse email et numéro de téléphone de la personne à contacter en cas de problème).

Tampon du service si disponible faisant apparaître au minimum Nom/Prénom/adresse/N° téléphone

BENEFICES ATTENDUS DE LARECHERCHE

- Bénéfice personnel thérapeutique attendu : En raison d'une neuropathie* induite par l'oxaliplatine, le traitement de chimiothérapie* peut être réduit, reporté ou complètement interrompu. Cela peut induire une diminution de l’action des traitements. Les patients qui seront dans le groupe PHYCOCARE pourront bénéficier potentiellement d’une prise en charge préventive de leur neurotoxicité* comparés aux patients du groupe PLACEBO*.

- Bénéfice collectif attendu : La participation volontaire à une recherche interventionnelle est utile à tous. Notre hypothèse, si elle est validée, devrait permettre de prévenir/diminuer la survenue de neurotoxicités* des patients traités par oxaliplatine.

ALTERNATIVES ALARECHERCHE

Si vous décidez de ne pas participer à cette étude, vous bénéficierez de la même prise en charge médicamenteuse pour traiter votre cancer gastro-intestinal métastatique.

FIN DE LARECHERCHE

La recherche peut être interrompue à tout moment :

- par les autorités de santé,
- du fait du promoteur*, le CHU de Nantes : si un élément nouveau survient, l’investigateur en sera informé et il vous transmettra alors les éléments susceptibles de modifier votre participation.
- du fait de l’investigateur, pour des raisons médicales vous concernant ; il peut décider à tout moment d’arrêter l’administration du produit à l’étude (par exemple à cause d’un effet indésirable ou d’une évolution de votre état de santé) et vous en informera.

Quelle que soit la raison de l’interruption, l’investigateur vous informera alors des mesures à suivre et vous continuerez à être suivi pour votre cancer gastro-intestinal métastatique.

Si vous décidez de participer à cette recherche, il s’agira d’un acte volontaire. Vous pourrez à tout moment décider d’arrêter votre participation, sans pénalité ni préjudice. Dans ce cas, vous devez informer l’investigateur de votre décision.

Quelle que soit la raison de votre interruption, l’investigateur vous informera alors des mesures à suivre.

Dans tous les cas, la qualité de votre prise en charge ne sera pas diminuée.

FRAIS SUPPLEMENTAIRES

Votre participation à cette recherche n'engendrera pour vous aucun frais supplémentaire par rapport à ceux que vous auriez dans la prise en charge habituelle de cette maladie.

VOS DROITS PENDANT LARECHERCHE

❖ SECRET PROFESSIONNEL

Le personnel impliqué dans la recherche est soumis au secret professionnel, tout comme votre médecin traitant. Sauf avis contraire de votre part, votre médecin traitant pourra être informé de votre participation.

❖ TRAITEMENT DE VOS DONNEES – ACCES AUX DONNEES VOUS CONCERNANT - CONFIDENTIALITE

• FONDEMENTS JURIDIQUES DU TRAITEMENT DES DONNEES

Le traitement de vos données personnelles a pour fondement juridique l’intérêt public que revêt cette recherche (article 6.1.e du Règlement (UE) 2016/679 du Parlement européen et du Conseil du 27 avril 2016 relatif à la protection des personnes physiques à l'égard du traitement des données à caractère personnel et

à la libre circulation de ces données (RGPD)). Il s'agit d'une des exceptions prévues par l'article 9 du même texte, permettant le traitement des données de santé.

Le traitement est conforme à la méthodologie de référence MR001 publiée par la CNIL.

- **TRAITEMENT DE VOS DONNEES**

Dans le cadre de cette recherche, un traitement informatique de vos données personnelles va être mis en œuvre : cela permettra d'analyser les résultats de la recherche et de remplir l'objectif de la recherche.

Pour cela, les données recueillies vous concernant seront des données de santé.

Ces données issues de votre dossier médical et des questionnaires ONLS et de qualité de vie EORTC-QLQC30 seront recueillies dans un cahier électronique (eCRF) auquel aura accès le Promoteur de la recherche (CHU de Nantes). Des données de suivi pourront également être recueillies au-delà de la stricte durée de l'étude.

- **CONFIDENTIALITE**

Afin d'assurer leur confidentialité, vos données seront identifiées par un numéro de code et vos initiales.

Les personnes intervenant dans la recherche dans le cadre de leurs missions auront accès à vos données codées.

Les seules personnes qui auront accès à vos données directement identifiantes sont les personnes qui assurent votre prise en charge dans le cadre de la recherche et les personnes responsables du contrôle et de l'assurance qualité de la recherche.

Vos données pourront, dans des conditions assurant leur confidentialité, via des plateformes ou serveurs sécurisés, faire l'objet de transferts nationaux ou internationaux (comme, par exemple, aux personnes ou sociétés agissant pour le compte du CHU, aux autorités sanitaires habilitées) pour cette étude, ou pour d'autres recherches ultérieures, exclusivement à des fins scientifiques.

Au sein de l'Europe la protection de vos données est garantie (Règlement européen UE 2016/679). Hors Europe vos données pourront être transmises dans des états n'ayant pas le même niveau d'exigence en termes de protection des données. Le cas échéant, le Promoteur prendra toutes les mesures nécessaires pour protéger les données recueillies et devra s'engager à assurer un niveau de sécurité équivalent à celui couvert par les lois françaises et européennes pour les données envoyées à l'étranger. Si vous le souhaitez, vous pouvez obtenir la copie de l'ensemble des mesures prises pour assurer l'ensemble de la protection de vos données auprès du Délégué à la Protection des Données (DPD) voir en début de document, paragraphe « vos contacts ».

Vos données pseudonymisées (identifiées par un numéro de code et vos initiales) seront susceptibles d'être exploitées dans le cadre de publications ou de communications ; dans ce cas votre anonymat sera préservé.

- **VOS DROITS**

Conformément aux dispositions de la loi relative à l'informatique aux fichiers et aux libertés (loi modifiée du 6 janvier 1978) et du Règlement (UE) 2016/679 du Parlement européen et du Conseil du 27 avril 2016 relatif à la protection des personnes physiques à l'égard du traitement des données à caractère personnel et à la libre circulation de ces données (RGPD), vous disposez d'un droit d'accès, de rectification, de limitation et d'opposition du traitement de vos données personnelles. Si vous décidez de retirer votre consentement pour participer à la recherche, les données obtenues avant que celui-ci n'ait été retiré seront utilisées conformément à l'article L.1122-1-1 du CSP. Les données recueillies après le retrait de votre consentement ne seront pas utilisées pour cette recherche et resteront destinées à l'usage strict du soin. Vous pouvez également porter une réclamation auprès d'une autorité de contrôle (CNIL pour la France : https://www.cnil.fr/fr/webform/adresser-une-plainte/).

Ces données pourront être utilisées lors de recherches ultérieures exclusivement à des fins scientifiques. Vous pouvez retirer votre consentement à cette utilisation ultérieure ou exercer votre faculté d'opposition à tout moment.

Vous pouvez également accéder directement ou par l'intermédiaire d'un médecin de votre choix à l'ensemble de vos données médicales en application des dispositions de l'article L 1111-7 du Code de la Santé Publique.

- **Devenir de vos donnees**

Vos données seront conservées tout au long de la recherche. Après la fin de la recherche, les données seront archivées pour une durée conforme aux dispositions réglementaires (soit au moins 15 ans après la fin de la recherche), puis détruites.

Pour en savoir plus ou exercer vos droits concernant vos données, voir en début de document, paragraphe « vos contacts ».

❖ **Acces aux resultats globaux de la recherche**

A la fin de la recherche, et à votre demande, vous pourrez être informé(e) par l'investigateur des résultats globaux de cette recherche (dès qu'ils seront disponibles).

LE CADRE REGLEMENTAIRE

Cette recherche est conforme :

- Aux articles L. 1121-1 à L. 1126-12 du Code de la Santé Publique relatifs aux recherches impliquant la personne humaine
- A la loi « Informatique et Libertés » du 6 janvier 1978 modifiée et la loi n° 2018-493 du 20 juin 2018 relative à la protection des données personnelles
- au Règlement (UE) 2016/679 du Parlement européen et du Conseil du 27 avril 2016 relatif à la protection des personnes physiques à l'égard du traitement des données à caractère personnel et à la libre circulation de ces données (RGPD)

Vous pouvez retrouver tous ces textes sur le site http://www.legifrance.gouv.fr

Conformément aux dispositions réglementaires :

➢ Le CHU de Nantes organise cette recherche en tant que « promoteur ». Il a souscrit un contrat d'assurance garantissant sa responsabilité civile et celle de tout intervenant auprès de la compagnie HDI Global SE (Contrat n° 0100775930012 210009).

➢ Cette recherche a reçu l'avis favorable du Comité de Protection des Personnes Ile de France VIII le30/09/2021.

➢ La recherche a aussi reçu l'autorisation de l'ANSM (Agence Nationale de Sécurité du Médicament et des Produits de Santé), le 23/08/2021

Conformément aux recommandations du Plan Cancer III (Action 5.4.), ce document a été soumis pour relecture, avis et conseil au Comité de Patients pour la Recherche Clinique en Cancérologie de la Ligue nationale contre le cancer.

EN RESUME…

Votre participation à cette recherche est libre. Vous pouvez refuser de participer à cette étude.

De plus, vous pouvez à tout moment vous retirer de cette recherche, sans préjudice.

Si vous décidez de refuser de participer à la recherche ou si vous décidez d'arrêter votre participation pendant la recherche :

- **cela n'aura aucune conséquence sur la qualité des soins qui vous seront donnés,**
- **vous devez simplement en informer l'investigateur.**

Lorsque vous aurez lu cette note d'information et obtenu les réponses aux questions que vous vous posez en interrogeant l'investigateur, il vous sera proposé, si vous en êtes d'accord, de donner votre consentement écrit en signant l'attestation préparée à cet effet.

Votre participation à la recherche, au cas où vous donneriez votre accord, ne pourra vous être confirmée qu'à la condition que vous remplissiez tous les critères d'inclusion pour participer à cette recherche.

Si le nombre de personnes prévues dans l'étude a été atteint, vous pourriez finalement ne pas être inclus dans l'étude même après avoir signé votre consentement. Dans ce cas précis, vous ne serez plus suivi dans le cadre de l'étude et les données vous concernant seront détruites. Cela ne portera, en aucun cas, préjudice sur la qualité de votre prise en charge médicale.

Vous pouvez prendre le temps nécessaire (quelques jours) avant de nous donner votre réponse.

**Au cours de ce délai de réflexion, vous pouvez bien entendu continuer par téléphone à poser toutes les questions que vous souhaitez à l'investigateur (Dr/Pr/Mme/M……………………..
tél………………………..).**

Nous vous prions d'agréer, Madame, Monsieur, l'expression de nos sentiments les plus respectueux.

Dr Le GOUILL-JAIJARAT Christèle, médecin coordonnateur et investigateur principal,

et toute l'équipe médicale en charge de cette recherche

Vous devez conserver un exemplaire de ce document.

ANNEXE 1 : CALENDRIER DES VISITES DE LARECHERCHE

Actions	Visite Inclusion J-30/J-4	Avant Cycle 1	Cycle 1 à cycle 12 (au maximum)		Cycles 13 à 18		Visite de fin d'étude (28 jours après la dernière prise de PHYCOCARE/ PLACEBO
		J-3 à J-1 (à domicile)	Jours de chimiothérapie	Autres jours	Jours de chimiothérapie	Autres jours	
Information patient Consentement éclairé	X						
Antécédents	X						
Examen clinique	X		J1 de chaque cycle de chimiothérapie		J1 de chaque cycle de chimiothérapie	Avant chaque attribution d'une nouvelle boîte de traitement	X
Questionnaire sur les neuropathies (ONLS)	X		à M4 (le jour du Cycle 9 jour 1)				X
Questionnaire sur la qualité de vie (EORTC- QLQ-C30)	X		à M4 (le jour du Cycle 9 jour 1)				X
Electromyogramme	X		à M4 (le jour du Cycle 9 jour 1)				X
RANDOMISATION	X						
PHYCOCARE / PLACEBO		X		Une fois par jour		Une fois par jour les jours sans chimiothérapie	
Carnet de prise de traitement		X		X		X	
Récupération de la nouvelle boite de traitement			Tous les 28 jours (un cycle sur deux)				
Retour du carnet complété			Une fois par mois, lors de la récupération d'une nouvelle boite de traitement				
Relevé des Evénements indésirables		X	X	X	X	X	X

CENTRE HOSPITALIER UNIVERSITAIRE DE NANTES<br>Version n° 2.0<br>Date : 23/05/2022	**Attestation de consentement à une recherche**<br>**« Étude randomisée, en double aveugle, multicentrique, contrôlée par placebo, évaluant la neurotoxicité chez des patients atteints d'un cancer gastro-intestinal métastatique prenant du phycocare ou un placebo pendant une chimiothérapie* à base d'oxaliplatine»**<br>**Titre abrégé : « PROPERTY»**<br>**Promoteur *: CHU de Nantes***<br>**Ref : RC21_0246 *N° EudracT / n° d'enregistrement :*** 2021-A01568-33

Je soussigné(e)
M^{e}, M. *(rayer la mention inutile*) (*prénom*, NOM) …………………………………………………………………………

Date de naissance : …../……/…….. **accepte librement et volontairement de participer à la recherche référencée ci-dessus,** coordonnée par le Docteur Le Gouill-Jaijarat Christèle et organisée par le CHU de Nantes, promoteur de la recherche.

Etant entendu que :

- L'investigateur qui m'a informé(e) et a répondu clairement à toutes mes questions, m'a précisé que ma participation est libre et que je peux me retirer de la recherche à tout moment.
- J'atteste ne pas faire l'objet de mesure de protection (tutelle, curatelle, sauvegarde de justice), en outre je confirme être affilié(e) ou bénéficier d'un régime de sécurité sociale.
- Il m'a été préalablement remis une note d'information sur cette recherche précisant son but, sa méthodologie, ses bénéfices attendus et ses risques prévisibles.
- Je pourrai avoir communication par l'investigateur, au cours ou à l'issue de la recherche, des informations qu'il détient concernant ma santé.
- Je sais que je peux retirer à tout moment mon consentement à ma participation à cette recherche et cela quelles que soient mes raisons et sans supporter aucune responsabilité, mais je m'engage dans ce cas à en informer l'investigateur. Le fait de ne plus participer à cette recherche ne portera pas atteinte à mes relations avec cet investigateur, ni à la qualité des soins qui me seront donnés.
  - J'accepte que mon médecin traitant soit informé de ma participation à la recherche :

    Oui, j'accepte Non, je refuse
  - Je pourrai à tout moment demander des informations complémentaires à l'investigateur.
  - Si je le souhaite, à son terme, je serai informé(e) par l'investigateur des résultats globaux de cette recherche.
- Mon consentement ne décharge en rien l'investigateur et le promoteur de l'ensemble de leurs responsabilités et je conserve tous mes droits garantis par la loi.
- Je ne pourrai pas participer à une autre recherche interventionnelle pendant toute ma participation à cette recherche, et jusqu'à 28 jours après la dernière prise de traitement à l'étude
- J'accepte que les données enregistrées à l'occasion de cette recherche puissent faire l'objet d'un traitement informatisé par le promoteur ou pour son compte, et j'atteste avoir été informé de tous mes droits concernant mes données personnelles selon les modalités décrites dans la note d'information en vigueur, qui m'a été transmise pour ce protocole.

**PERSONNE SE PRETANT A LA RECHERCHE**		
**Date : ...... / ..... / ……….**	**Signature :**	
Le cas échéant : Attestation du consentement en cas d'impossibilité d'expression écrite de la personne qui se prête à la recherche par la personne de confiance, un membre de la famille ou un des proches		
Date : ...... / ..... / ………..	Prénom NOM : Lien avec la personne de prêtant à la recherche :	Signature :
**INVESTIGATEUR : J'atteste avoir pleinement expliqué à la personne signataire le but, les modalités ainsi que les risque potentiels de la recherche**		
**Date : ...... / ..... / ……….**	**NOM :**	**Signature :**

Ce document est à réaliser en 2 exemplaires originaux : le premier doit être conservé par l'investigateur et le deuxième est remis à la personne donnant son consentement. En cas de duplicata, l'original est conservé par l'investigateur et une copie est remise à la personne ayant donné son consentement. En cas de triplicata, le promoteur récupèrera un des duplicata des consentements dans des enveloppes scellées tout au long de l'étude.
